# Host Serine Proteases: A Potential Targeted Therapy for COVID-19 and Influenza

**DOI:** 10.3389/fmolb.2021.725528

**Published:** 2021-08-30

**Authors:** Yalda Rahbar Saadat, Seyed Mahdi Hosseiniyan Khatibi, Sepideh Zununi Vahed, Mohammadreza Ardalan

**Affiliations:** Kidney Research Center, Tabriz University of Medical Sciences, Tabriz, Iran

**Keywords:** coronavirus, COVID-19, human proteases, TMPRSS2, furin, influenza

## Abstract

The ongoing pandemic illustrates limited therapeutic options for controlling SARS-CoV-2 infections, calling a need for additional therapeutic targets. The viral spike S glycoprotein binds to the human receptor angiotensin-converting enzyme 2 (ACE2) and then is activated by the host proteases. Based on the accessibility of the cellular proteases needed for SARS-S activation, SARS-CoV-2 entrance and activation can be mediated by endosomal (such as cathepsin L) and non-endosomal pathways. Evidence indicates that in the non-endosomal pathway, the viral S protein is cleaved by the furin enzyme in infected host cells. To help the virus enter efficiently, the S protein is further activated by the serine protease 2 (TMPRSS2), provided that the S has been cleaved by furin previously. In this review, important roles for host proteases within host cells will be outlined in SARS-CoV-2 infection and antiviral therapeutic strategies will be highlighted. Although there are at least five highly effective vaccines at this time, the appearance of the new viral mutations demands the development of therapeutic agents. Targeted inhibition of host proteases can be used as a therapeutic approach for viral infection.

## Highlights

• Furin and TMPRSS2 mediate SARS-corona virus infection, SARS-CoV-2, and influenza entry into the human cells.
• As a causative factor, TMPRSS2 exerts more severe outcomes for COVID-19.
• Targeted inhibition of TMPRSS2 and furin may be used as a therapeutic approach for COVID-19.
• Bromhexine hydrochloride may be an effective therapeutic drug for COVID-19.

## Introduction

Over the last 2 decades, several outbreaks of coronaviruses (CoVs) have received worldwide attention since they were responsible for the SARS (severe acute respiratory syndrome coronavirus) in China (2002–2003) and the MERS-CoV (Middle East respiratory syndrome) in Saudi Arabia (2012). Currently, the world is struggling with the novel CoV (SARS-CoV-2), initially recognized in China in late 2019. Since its discovery, SARS-CoV-2 has spread globally and its epidemic disease (COVID-19) has claimed thousands of lives. To date, the high number of infected cases with severe respiratory illnesses and viral pneumonia is observed as a risky and rapid human-to-human transmission occurance (Ashour et al., 2020).

Receptor recognition is a significant element of SARS-CoV-2 infection, pathogenesis, determining host range, and a therapeutic target (Wang et al., 2020a; Shang et al., 2020). SARS-COV-2-spike S protein (SARS-COV-2-S), similar to SARS-CoV, exploits human angiotensin-converting enzyme 2 (ACE2) for its entry. Using the same entrance receptor as SARS-CoV, the same set of cells can be targeted and infected by SARS-CoV-2 (Rabi et al., 2020). *In situ* analysis of different tissues has revealed top primary vulnerable cells to SARS-CoV-2, including the AT2 lung cells and macrophages, adrenal gland stromal cells, cardiomyocytes, thyroid, ovary, and stromal testis cells. Some other cells are less likely to be the main targets of SARS-CoV-2 (including enterocytes, cholangiocytes, and the kidney proximal tubule cells) (Zhou et al., 2020).

Although binding to host cells is the initial step of infection, virus entrance necessities the cleavage of S protein via host proteases including cell surface transmembrane protease/serine (TMPRSS) proteases, cathepsins, furin, elastase, factor Xa, and trypsin (Lambertz et al., 2019; Ji et al., 2020; Liu et al., 2020; Luan et al., 2020). Evidence suggests that numerous respiratory viruses hijack host proteases in order to enhance their spread in the host body. Based on the accessibility of cellular proteases needed for SARS-S activation, SARS-CoV-2 entrance and activation can be mediated by two distinct ways; 1) by endocytosis and the cutting of the SARS-COV-2-S by cathepsin L in endosomes (Glowacka et al., 2011; Shirato et al., 2013) and 2) by TMPRSS2 provided that it is co-expressed with ACE2 on the target cells’ surface (Heurich et al., 2014). This binding process entails several conformational alternations in the viral envelope glycoproteins (Shen et al., 2017), resulting in virion internalization. Since SARS-CoV-2 employs host proteases as its entrance activators, their inhibitors may exert therapeutic benefits against COVID-19 (Bestle et al., 2020a) and SARS-CoV infections (Shrimp et al., 2020). In the present review, we will highlight the recent updates on the functional role of host proteases, specially TMPRSS2 and furin in viral infection and discuss the possible interventions for inhibiting these enzymes.

## SARS-CoV-2 Virion

**FIGURE 1:**
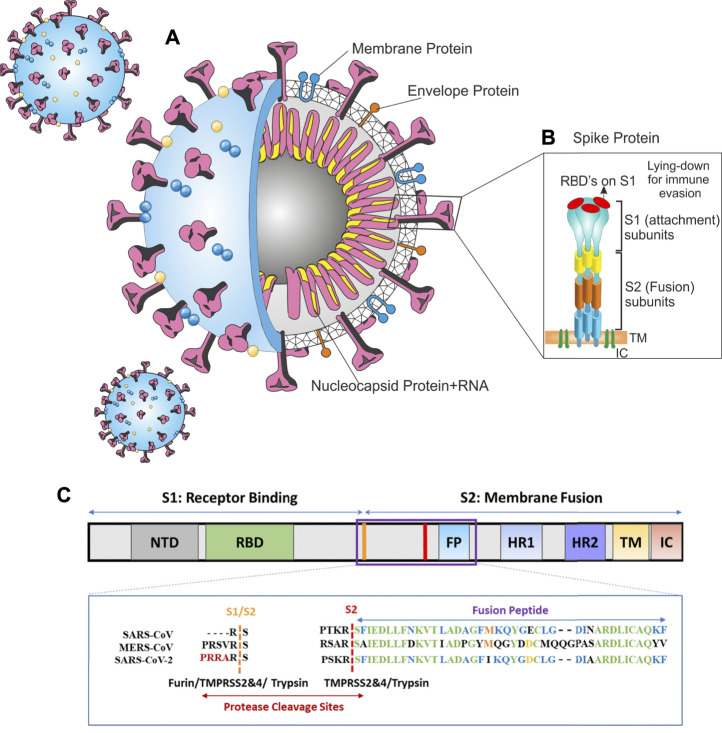
Architecture of SARS-CoV-2 virus **(A)** SARS-CoV-2 schematic **(B)** spike protein, **(C)** alignment of SARS-CoV-2, MERS-CoV, and SARS-CoV sequences in protease cleavage sites S1/S2 and S2. A furin cleavage motif (RRAR) only exists in the SARS-CoV-2 spike. Trypsin, furin, and TMPRSS2 and 4 can cleave the S1/S2 site within the receptor binding domain (RBD) of S protein generating the optimal conformation for viral binding to the host ACE2 receptor. The viral membrane fusion with the host membrane S2 domain can be cleaved by TMPRSS 2 and 4. Panel C is adapted from Luan et al. (2020).

The SARS-CoV-2 virion is a non-segmented RNA-positive virus (Zumla et al., 2016). Its genome encodes nucleocapsid (N), membrane (M), envelope (E), and spike (S) structural proteins. The M and E proteins are located among the spike proteins in the viral envelope (Figure 1A). The virion has a nucleocapsid composed of a single-stranded and positive-sense RNA in a size of 29.9 kb and highly immunogenic phosphorylated N-protein that is covered by the S and the hemagglutinin-esterase (HE) spike proteins, buried inside the phospholipid bilayers (Wu et al., 2020a; Jin et al., 2020).

The SARS-CoV-2 genome has a highly similar identity to the human SARS-CoV (80%) (Matsuyama et al., 2010), hence, they have analogous pathogenesis and biochemical interactions. The S protein eases receptor-binding and viral entrance into the target cells by the fusion of the viral and host cell membranes; hence, it can determine the host range (Heurich et al., 2014; Hoffmann et al., 2020a). Moreover, the spike S is the common target for vaccines and neutralizing antibodies. Spike protein has two functional subunits; the surface unit (S1) and S2 (Figure 1B). The S1 recognizes a cellular receptor by a receptor-binding domain (RBD), mediating viral attachment to the host ACE2. The S2 subunit has other basic features needed for the fusion of the viral and cell membranes. The high cleavable properties of the S1/S2 cleavage site of SARS-2-S are attributed to its multi-basic structure (several arginine residues).

In the CoVs, the RBD can be in a standing-up or a lying-down state. The first state permits receptor binding; however, the second state does not (Yuan et al., 2017). SARS-CoV-2-S is frequently in the lying-down state in comparison to SARS-CoV; consequently, despite having high affinity, it is less available to the ACE2. This difference results in a lower or comparable receptor binding affinity for the SARS-CoV-2 whole spike (Shang et al., 2020). The SARS-CoV-2’ RBD has a greater affinity to bind to the ACE2 than SARS-CoV; conversely, the whole spike glycoprotein of SARS-CoV-2 cannot bind to human ACE2 stronger than SARS-CoV’ spike (Shang et al., 2020). To preserve its RBD less accessible while retaining its high infectivity, SARS-CoV-2 depends on a second strategy; the activation of host proteases (Figure 1C).

## Entry and Priming of SARS-CoV-2 by the Host Proteases

**FIGURE 2:**
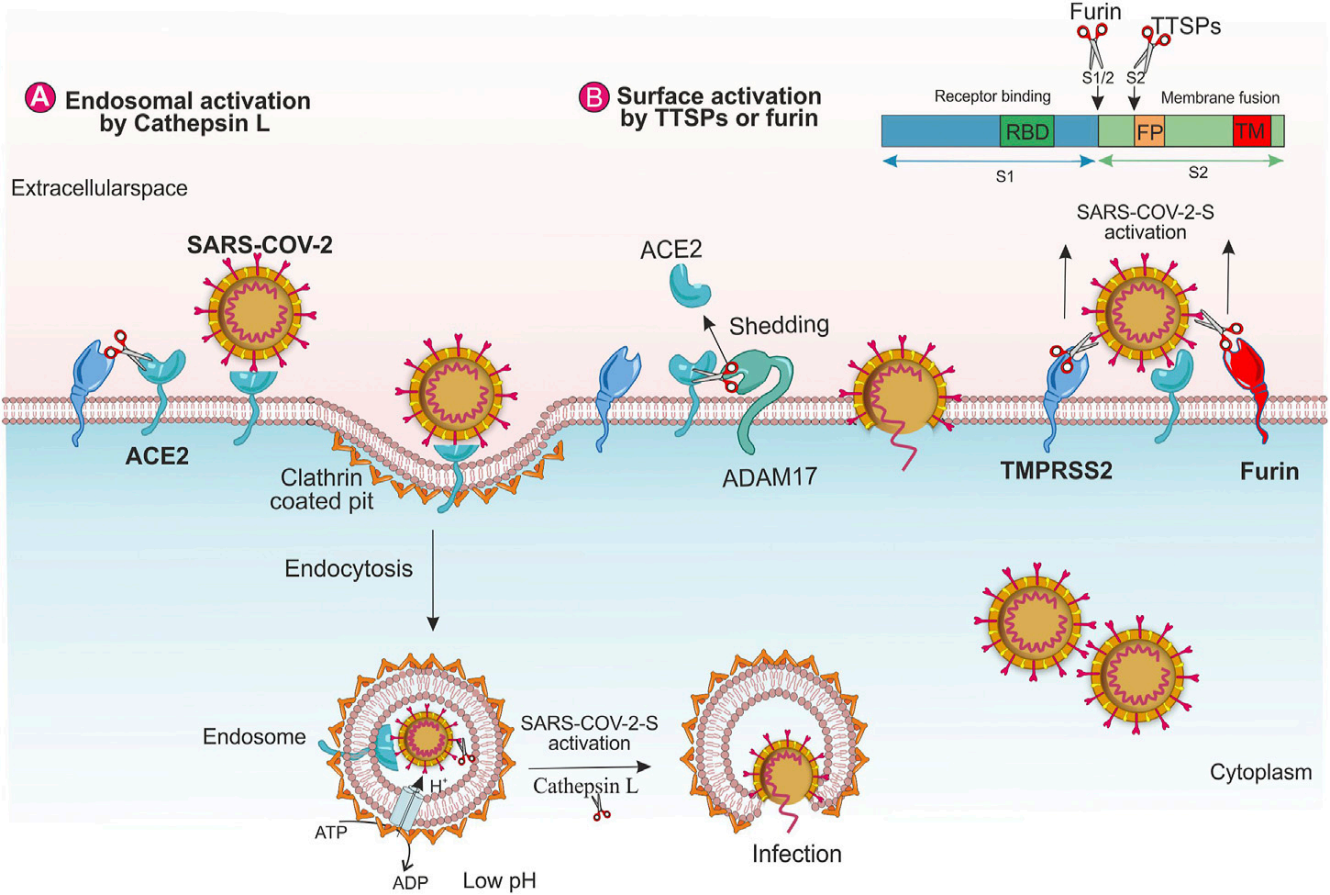
The role of human host proteases on SARS-CoV-2 entry. Virus entry through **(A)** endosomal pathway and **(B)** TMPRSS2 and furin.

The high binding affinity of SARS-CoV-2 to the human receptor is one reason why it is more hostile than SARS-CoV (Wu et al., 2020a; Lu et al., 2020). The viral infectivity highly relies on the cleavage of the S protein by the cell proteases that happens during different steps in the viral life cycle (Heurich et al., 2014). Two cellular proteolytic systems were utilized by SARS-CoV in order to guarantee the adequate processing of viral S protein (Figure 2). The SARS-CoV-2 interaction with the human cells is similar to SARS-CoV (Ragia and Manolopoulos, 2020). The proteolysis of the two peptide bonds (i.e., Arg685-Ser686) results in the so-called S1 and S2 subunits separation and the subsequent activation/priming of the S protein of SARS-CoV-2 (Fuentes-Prior, 2021). The presence of receptor-binding domain (RBD) in the N-terminal domain (NTD) of the S1 subunit causes direct binding to the peptidase domain of ACE2, whereas, the C-terminal of the S2 subunit is responsible for its attachment viral envelope after proteolysis at the S1/S2 site and eventually leads to its fusion with the host cell membrane (Benton et al., 2020; Ragia and Manolopoulos, 2020). Furin-mediated proteolysis results in the pre-activation of the S proteins in the SARS-CoV-2 virions besides entailing the cleavage of a single bond, Arg815-Ser816, for fusion machinery activation. The abovementioned exclusive properties of SARS-CoV-2 are indispensable for S protein-mediated cell–cell fusion and human cell entry (Fuentes-Prior, 2021). Additionally, early priming of the S protein depends on human TMPRSS2, which is necessary for SARS-CoV-2 entry (Ragia and Manolopoulos, 2020).

The binding of the SARS-S to the ACE2 can promote the virions endocytosis. It is reported that Cathepsin L cleaves the S protein of SARS-CoV-2 functionally and promotes viral entry (Zhao et al., 2021). SARS-S/ACE2 complex generates conformational changes in SARS-S, which in turn, may raise the sensitivity of the spike to proteolytic enzymes. Inside the endosome, Cathepsin L, a pH-dependent endosomal or lysosomal protease, facilitates the cleavage of SARS-S and activates the S protein for fusion within the endosomal membrane. During endosome maturation, the low pH of the endosomal environment activates the re-arrangement of HA that exposes the viral fusion peptide into the endosomal membrane (Shen et al., 2017; Simmons et al., 2013), (Figure 2A). Moreover, SARS-CoV-2 induces the transcription and enzyme activity of Cathepsin L, which, in turn, elevates viral infection (Chandran et al., 2005; Zhao et al., 2021).

## Host Cell Proteases; General Definition

The SARS-CoV-2’s spike protein has cleavage sites for the host cell proteases guaranteeing the exposure of the fusion sequences and viral entry. It is observed the that lysosomal and cell surface proteases can both activate SARS-CoV-2 entrance. Furthermore, other proteases such as furin have accumulative impacts on the entrance of SARS-CoV-2 (Shang et al., 2020).

### Furin

Furin and furin-like proteases are members of the proprotein convertases family. Furin, a kind of proprotein convertase, is a type I transmembrane protein expressed in all eukaryotic cells. It is activated by acid pH in the trans-Golgi network (Feliciangeli et al., 2006). Furin plays a key role in the cleavage of a wide range of critical cell surface proteins including adhesion molecules, surface receptors, growth factors, and hormones to produce mature proteins. Additionally, furin cleaves envelope glycoproteins of different viruses, thus, improving the fusion of viral membrane with the host cell (Garten, 2018). Data indicate that the existence of a redundant furin cleavage site at S protein of SARS-CoV-2 is responsible for its infectious nature than other CoVs, leading to its higher efficiency to fuse to the host membrane (Wu et al., 2020b).

### TMPRSS2

TTSPs, a group of membrane serine proteases, occupy a vital role in numerous physiological procedures (Antalis et al., 2010; Antalis et al., 2011; Hoffmann et al., 2018). These enzymes are characterized by an extracellular C-terminal domain with the serine protease activity, a single transmembrane domain, and a short cytoplasmic N-terminal domain. Böttcher et al. considered the TTSPs as the activators of viral infection. They indicated that the TMPRSS2 and the HAT (a trypsin-like protease) result in the activation of the FLUAV-HA and the spread of the FLUAV in the infected host (Hoffmann et al., 2018). TMPRSS2 is a single-pass cell membrane-anchored TTSP protein with 492 amino acids expressed on epithelial cells of some tissues and found to regulate cell-matrix and cell-cell interactions. TMPRSS2 expression seriously stimulates the replication and syncytium formation of coronaviruses *in vitro* and *in vivo* (Matsuyama et al., 2010; Shulla et al., 2011; Shirato et al., 2013; Simmons et al., 2013; Iwata-Yoshikawa et al., 2019a), exerting an important role in their spread (Iwata-Yoshikawa et al., 2019b). Furthermore, the TMPRSS2 might stimulate viral pathogenesis and spread *via* activating spike S for virus-cell and cell-cell fusion and neutralizing antibodies that decrease viral recognition (Glowacka et al., 2011).

### Cathepsins

Human cathepsins are endosomal proteases with broad proteolytic activity in acidic pH. Cathepsins activation in lysosomes may exert an important role in SARS-CoV and MERS-CoV entry via endocytosis (Sahebnasagh et al., 2020). Lysosomal cathepsin L cleaves peptide bonds with hydrophobic residues in the P3 position and aromatic residues in the P2 position (Vargas-Alarcón et al., 2020). Cathepsin L incorporates in glycoprotein processing of SARS-CoV and Ebola (Pišlar et al., 2020; Vargas-Alarcón et al., 2020). Recent investigations assessed cathepsins’ role in SARS-CoV-2 entry. Further, the role of cathepsin L is highlighted in virion entry into human embryonic kidney 293 cells expressing ACE2; however, CA-074 did not significantly affect SARS-CoV-2 entry (Pišlar et al., 2020).

### Neutrophil Elastase

Neutrophils as a part of the host defense system, release a granular serine protease (the so-called elastase) in response to aviral infection (Thierry, 2020; Vargas-Alarcón et al., 2020). Increased elastase activity mediates acute lung injury through increasing inflammatory reactions (e.g., increasing vascular permeability, induction of pro-inflammatory cytokines such as IL-8 and IL-6 secretion by neutrophil vesicles, and conversion of pro-IL-1β to IL-1β) (Bai et al., 2020). Under normal conditions, the NE’s function is regulated by the inhibitors of endogenous protease. Nevertheless, under pathophysiological conditions, neutrophil oxidants inactivate the aforementioned inhibitors which result in hydrolyzing the host extracellular matrix proteins such as collagen-IV as well as elastin and subsequent damage to the endothelial barrier and infiltration into the bronchoalveolar space (Korkmaz et al., 2020; Thierry, 2020).

### Plasmin

Pathogens, especially viruses, convert plasminogen to plasmin in order to cleave surface proteins and subsequently can evade the immune system or infect host cells (Medcalf et al., 2020). Plasmin can cleave new furin sites in SARS-CoV S protein which in turn results in increased viral infectivity (Ji et al., 2020). In addition to the contribution of early stages of a viral infection, plasmin can provoke cytokine production and stimulate inflammation *via* factor XII/bradykinin which subsequently can increase edema (Medcalf et al., 2020). The elevated levels of plasmin (ogen) were observed in COVID-19 patients (Ji et al., 2020). Plasmin can cleave hyaline membranes (consist of a fibrin network combined with serum proteins and cellular debris, acts as barriers to gas exchange in the alveoli), which is considered asa histopathological hallmark of acute respiratory distress syndrome (ARDS) induced by SARS-CoV-2. In patients affected by the ARDS, bronchoalveolar lavage samples showed elevated levels of active plasmin and plasminogen (Henry et al., 2020).

## The Functional Role of Host Proteases in SARS-CoV-2 Infection

### The Role of Furin in SARS-CoV-2 Infection

Bioinformatics analysis on the SARS-CoV-2-S sequences has anticipated a novel polybasic furin cleavage site containing an insertion of amino acid residues (–PRRA–) between S1 and S2 subunits (Figure 1C). Based on the genomic characteristics of SARS-CoV-2, furin can cleave the viral spike protein at that site and activate it (Mallapaty, 2020). Coutard et al. identified a furin-like cutting site in the SARS-CoV-2-S which is not available in other SARS-like CoVs such as Pangolin, Bat-CoV, and SARS-CoV-1. MERS has a pseudo-furin- binding site (Coutard et al., 2020; Vankadari, 2020). Likewise, Vankadari found that structurally, furin interacts with the SARS-CoV-2-S that highlighted the mechanism of viral host cell entry (Vankadari, 2020).

In lung cells that highly express furin and fail to express strong cathepsin L levels, pre-cleavage of the S proteins by furin is needed for consequent activation of spike protein by TMPRSS2 in both MERS-CoV and SARS-CoV-2 (Hoffmann et al., 2020b). In order to initiate the membrane fusion of viral and human cells along with the passage of viral genome into the cytoplasm of the host cell, viral spike S glycoprotein needs to be sequentially cleaved at S1/S2 and S2’ “sites. Furin cleaves the S1/S2 site, while the TMPRSS2 processes at the S2” site, and these enzymes cannot compensate for each other. Finally, at the Golgi or endoplasmic reticulum compartment, nascent viruses are assembled and released from the infected cells by exocytosis (Hoffmann et al., 2018). Preactivation of furin permits SARS-CoV-2 to be less reliant on host cells, improving its entry into some target cells that relatively express low levels of lysosomal cathepsins and/or TMPRSS2 (Shang et al., 2020).

It is revealed that the furin enzyme is expressed in other potential target organs of the coronavirus such as the intestine, colon, ileum, rectum, heart, and oral mucosa tissues (Dittmann et al., 2015; Mei et al., 2020). The furin universal expression in some tissues and organs may be an explanation for the high pathogenicity and transmissibility of SARS-CoV-2 (Wang et al., 2020a). Therefore, in the course of SARS-CoV-2 infection, the presence of furin may result in some clinical symptoms (Dittmann et al., 2015), for example, the furin-mediated entrance of SARS-CoV-2 into the cardiomyocyte may clarify the cardiac injury in patients with COVID-19 (Dittmann et al., 2015). Moreover, the furin protease activity in the oral mucosa tissues makes them susceptible to SARS-CoV-2 and may be associated with oral symptoms in COVID-19 like taste blindness and dry mouth (Mei et al., 2020).

### The Functional Role of TMPRSS2 in Viral Infection

Accumulating evidence reveals that SARS-CoV-2 and relevant viruses such as MERS-CoV, influenza A virus (FLUAV), and SARS-CoV require TMPRSS2 activity as a host cell factor for their spread (Hoffmann et al., 2020a; Kim J et al., 2020). Moreover, it is reported that the VeroE6 cell line, expressing TMPRSS2, is very vulnerable to SARS-CoV-2 infection; indicating that similar to MERS-CoV and SARS-CoV, SARS-CoV-2 infection is boosted by TMPRSS2 (Matsuyama et al., 2020). Furthermore, both lysosomal cathepsins and TMPRSS2 have accumulative effects with a calcium-dependent proprotein/prohormone convertase (furin) on activating the entrance of SARS-CoV-2 in some cells such as lungs. (Shang et al., 2020).

TMPRSS2, by cleaving the SARS-CoV-2-S facilitates virus entrance and activation (Hoffmann et al., 2020a). The co-expression of TMPRSS2 in ACE2-positive lung cells suggests that it exerts a critical role in the spread of the virus in the human respiratory tract (Heurich et al., 2014).

TMPRSS2 eases the infection of SARS-CoV by two independent mechanisms; a) by ACE2 cleavage that enhances the viral entry and stimulates viral uptake by cathepsin L-dependent entry, not activating SARS-S for entry (Figure 2A), b) by SARS-S cleavage at the host cell surface that activates the spike protein for membrane fusion (Figure 2B) (Heurich et al., 2014). Two amino acid residues (arginine and lysine) within the ACE2 (697–716 and 652–659) are critical for TMPRSS2 and the metalloprotease ADAM17, respectively. These enzymes compete together for the ACE2 cleavage; however, only TMPRSS2-mediated-ACE2 cleavage stimulates SARS S-driven entry (Shulla et al., 2011; Heurich et al., 2014). The ADAM17 eases the ACE2 shedding into the extracellular space and stimulates SARS-CoV uptake into the host cells (Figure 2B). Additionally, ADAM17 facilitates the release of the TNF-α (tumor necrosis factor-α) and IL-6 receptors. The TNF-α exerts autocrine and paracrine function and TNF-α/its receptor signaling elevates ADAM17 activity. Viral infection and endocytosed SARS-CoV-2 spike proteins also elevate ADAM17 activity. An increased ACE2 shedding by ADAM17 leads to the down-regulation of ACE2 that increases the angiotensin II levels, resulting in further rises in ADAM17 activity (Gheblawi et al., 2020).

Different residues (R667 and R797) in SARS-S control the TMPRSS2-mediated S activation indicating that these procedures are more complex than initially appreciated (Reinke et al., 2017). Glowacka et al. concluded that based on the location of the TMPRSS2, the processing of the SARS-S cleavage by host cell TMPRSS2 can have different results. In the secretory pathway of infected cells, when the TMPRSS2 is co-expressed with SARS-S in the same cell, spike cleavage leads to the shedding of SARS-S into the supernatants, and as antibody decoys, the S protein fragments inhibit antibody-mediated neutralization (Glowacka et al., 2011).

Evidence proposes that the TMPRSS2 may control the function of mitochondria through the estrogen-related receptor-α (ERR-α) (Xu et al., 2018). ERR-α, a nuclear receptor, along with its coactivator peroxisome proliferator-activated receptor-γ coactivator-1α (PGC-1α) regulate mitochondrial functions and energy homeostasis at the transcriptional level (Xu et al., 2018). Mitochondrial hijacking by SARS-2 may be one of the underlying mechanisms leading to COVID-19. Few human genes including a subunit of ubiquitin-protein ligase complex (FBXO21) and mitochondrial ubiquitin specific peptidase 30 (USP30) appear to be targeted by viral RNA (Pasquier and Robichon, 2020). During infection, viral RNAs can be translocated into the mitochondria to hijack and utilize host mitochondria. By modifying ubiquitination and impacting mitochondrial function, SARS-CoV-2 can repress host immunity in COVID-19 cases *via* different mechanisms. One possible viral mechanism can be mediated by regulating the host USP30. USP30 regulates the mitochondrial homeostasis and dynamics (fusion and fission). The presence of 20 nucleotides in open-reading frame 3a (ORF3a) of SARS-CoV-2 can target a sequence in mitochondrial USP30 transcripts (Pasquier and Robichon, 2020). Utilizing the host mitochondria by viral ORFs can result in mitochondrial DNA (mtDNA) release in the cytoplasm that activates the mtDNA-induced inflammation and represses both innate and adaptive immunity. Finally, the mitochondrial collapsing by virus results in the death of the infected cells (Singh et al., 2020).

#### The TMPRSS2 Gene

The *TMPRSS*2 gene consists of 14 exons and 13 introns (44 kb in length) and is located on human chromosome 21. It is more expressed in prostate cancer cells (Paoloni-Giacobino et al., 1997). One of the important characteristics of the *TMPRSS*2 gene is located at position −148 with several 15-bp androgen response elements (AREs) at the upstream of the transcription start origin (Lin et al., 1999; Afar et al., 2001). In prostate cancer cells, androgenic hormones up-regulate the *TMPRSS*2 gene which probably is mediated by the androgen receptor (Antalis et al., 2011; Shen et al., 2017; Ashour et al., 2020). Studies in knock-out murine models revealed that these mice are resistant to the spread and pathogenesis of some subtypes of the FLUAV. As mentioned earlier, TMPRSS2 can be regulated by androgen and its receptor (Chen et al., 2019) and the presence of AREs on the promoter of the *TMPRSS*2 gene may be the underlying cause of the severity and higher mortality of COVID-19 in men (Zununi Vahed et al., 2020). Moreover, the promoter of the human *TMPRSS*2 gene has a guanine-rich region forming G-quadruplex secondary structures that can block or reduce *TMPRSS*2 transcription in the presence of potassium ions (Shen et al., 2020). Furthermore, studies regarding the *TMPRSS*2 gene polymorphisms that cause *TMPRSS*2 gene overexpression in humans, showed its association with severe influenza (Hoffmann et al., 2018). It is demonstrated that genetic variation in TMPRSS2 including two identified SNP in *TMPRSS*2 (rs383510 and rs2070788) had a strong correlation with the A (H7N9) influenza susceptibility (Cheng et al., 2015).

Complete computational analyses indicated that functional single nucleotide polymorphisms of *TMPRSS*2 gene and epigenetic mechanisms play important roles in the diverse susceptibility of different populations to SARS-CoV-2 (Paniri et al., 2020). *TMPRSS*2 genetic variants including rs383510, rs2070788 37, rs469390, and rs464397 strongly increase the TMPRSS2 expression in lung tissue, where these variants at higher frequencies are present in European and American populations than the Asian populations. This result suggests these populations may be quite more vulnerable to SARS-CoV-2 infection (Irham et al., 2020).

#### TMPRSS2 Expression in Different Cells

Remarkably, the TMPRSS2 has a highly variable expression in humans and its expression may be positively associated with COVID-19 severity. The TMPRSS2 is mainly expressed in the epithelium cells of the prostate and has a vital role in its carcinogenesis (Lucas et al., 2008). Matsuyama et al. (2010) indicated an interaction of the TMPRSS2-expressing cells with viral tropism and pathogenicity of SARS-CoV infection. The TMPRSS2 is also expressed in the lungs (Bertram et al., 2012), digestive tract, kidney, the cardiac endothelium proposing that these organs may be essential targets for SARS-CoV-2 infection (Bertram et al., 2012). Indeed, the COVID-19 clinical manifestations include complications from gastrointestinal symptoms, higher liver enzymes, acute kidney injury (AKI), and acute myocardial damage. It was approved that the expression of the ACE2 and the TMPRSS2 provides SARS-CoV-2 entrance on the ocular surface cells. Moreover, the co-express of the TMPRSS2 and the ACE2 in the prostate epithelial cells may be involved in more pathogenicity of COVID-19 disease in males than females (Song et al., 2020). The TMPRSS2 and TMPRSS4 promote virus entry into the intestinal cells by facilitating SARS-CoV-2 spike fusogenic activity (Zang et al., 2020).

Based on the different datasets of gene expression, it is found that the expression levels of the TMPRSS2 and the ACE2 are significantly higher in the nasal epithelium in comparison to saliva and blood, where their levels decrease in lower airway tissues. Significantly, the expression levels of these genes in the bronchial and nasal tissues are lower in children than adults. The result of this study indicates that the severity of COVID-19 between adults and children, in part, can be attributed to the different expression levels of the TMPRSS2 and the ACE2 in airways tissues (Saheb Sharif-Askari et al., 2020).

Mechanisms that influence SARS-CoV-2 infectivity and clinical outcomes of COVID-19 are reported by analyzing the nasal airway transcriptome of children. In this study, it is found that the ACE2 expression is upregulated by interferon response to respiratory viruses. Moreover, the action of IL-13a upregulates the *TMPRSS*2 as a mucus secretory network gene (Sajuthi et al., 2020).

## Therapeutic Strategies to Target Host Proteases for COVID-19

Unraveling the viral RBD features can open a new horizon to block the spike cleavage sites and develop protease inhibitors. Recent studies suggest that host proteases are vital for the activation of the SARS-CoV-2 in human epithelial cells, hence, they can be hopeful drug targets for the management of COVID-19 (Bestle et al., 2020a). Therapeutic inhibition of the TMPRSS2 and furin may be used as a therapeutic approach for COVID-19. A list of host proteases inhibitors is listed in Table 1. In the following section, we review the potential drugs or compounds that may alter the TMPRSS2 expression and activity.

**TABLE 1:** A list of host protease inhibitors against COVID-19

Targets/Inhibitors	Mechanism of action	Studied models	References
Furin protease inhibitors			
MI-1851	-Inhibits virus entry by preventing furin cleavage at S1/S2 site of S protein -Inhibits SARS-CoV-2 replication	*In vitro*	Bestle et al. (2020a)
Diminazene (an anti-parasitic drug)	It occupies the substrate-binding pocket of furin.	*In vitro*	Wu et al. (2020b)
Decanoyl-RVKR-chloromethylketone (CMK)	CMK blocks SARS-CoV-2 entry, suppresses cleavage of spikes and the syncytium. Also, it affects the early stage of the virus replication cycle	*In vitro*	Cheng et al. (2020)
Naphthofluorescein	It suppresses SARS-CoV-2 RNA transcription rather than virus entry.	*In vitro*	Cheng et al. (2020)
TMPRSS2 protease inhibitors			
MI-432	Inhibits virus entry by hindering TMPRSS2 cleavage at S2 site of S protein	*In vitro*	Meyer et al. (2013)
MI-1900			
Aprotinin	-Inhibits virus entry by hindering TMPRSS2 cleavage at S2 site of S protein-Prevents double-stranded RNA formation in SARS-CoV-2 infected cells	*In vitro*	Bojkova (2020)
Excavatolide M, Dictyosphaeric Acid A, Durumolide K, Schisphenin ACytidine (5)-Diphosphocholine (Citicoline), 5-Methoxyhydnocarpin D Polyphenol (-)-Epicatechin 3-O-(30-O-Methyl) Gallate, Curtisian L, Microcarpin, Geniposide, NPC306344, Isogemichalcone B	These compounds interact with the active site residues of TMPRSS2 and inhibit it	In silico	Rahman et al. (2020)
Nafamostat mesylate	Prevents S-glycoprotein activation by inhibiting TMPRSS2	Clinical trial	
Camostat mesylate	-Decreases SARS‐S‐, MERS‐S‐, and SARS‐2‐S‐ significantly -Decreases authentic SARS‐CoV‐2 infection in the Calu‐3 lung cell line -Inhibits SARS‐S‐ and SARS‐2‐S entry into primary human lung cells	*In vitro*	Hoffmann et al. (2020a)
	Beyond its antiviral activity, camostat may decrease the uncontrolled cytokine storm observed in severe COVID-19, since the expression of TMPRSS2 is necessary for cytokine release upon exposure of mice to polyIC	*In vivo*	Iwata-Yoshikawa et al. (2019c)
	- when applied with inhibitor E-64d, completely blocks the SARS-2-S-driven entry -Partially blocks SARS-CoV-2-S and SARS-CoV-driven entry by inhibiting the TMPRSS2	Clinical trial	
Gabexate			
Bromhexine		Clinical trial	Ansarin et al. (2020)
Cathepsin B and L inhibitors			
CA‐074 (#HY‐103350): an inhibitor of Cathepsin B	Had no marked effect on virus entry		Sahebnasagh et al. (2020)
E64D: an endosomal cysteine proteases (CatB/L) inhibitor	Reduced entry of SARS‐CoV‐2 S pseudovirions	*In vitro*	Sahebnasagh et al. (2020)
	Had no effect on virus replication in Calu-3 (human airway epithelial cells)	*In vitro*	Bestle et al. (2020a)
	E63D interfere efficiently with SARS‐2‐S‐driven entry into the TMPRSS2-cell lines 293 T and Vero	*In vitro*	Hoffmann et al. (2020e)
Dalbavancin (a lipoglycopeptide antibiotic) :an inhibitor of Cathepsin L	By preventing cathepsin L in the late endosome/ lysosome represents an antiviral effect. in a dose‐dependent manner it could hinder the entry of SARS‐CoV‐2	*In vitro*	Zhang (2020)
Teicoplanin (a glycopeptide antibiotic): an inhibitor of Cathepsin L	Prevents HIV‐luc/2019‐nCoV‐S pseudoviruses entry in a dose‐dependent manner	*In vitro*	Zhang (2020)
SID 26681509 (#HY‐103353): an inhibitor of Cathepsin L	Reduced entry of SARS‐CoV‐2 S pseudovirions in 293/hACE2	*In vitro*	Sahebnasagh et al. (2020)
Other protease inhibitors			
BenHCl, an inhibitor of Factor Xa	Factor Xa could cleave the full-length recombinant S protein into S1 and S2 subunits, and this cleavage		Du et al. (2007)
Rivaroxaban, apixaban, edoxaban, andbetrixaban, inhibitors of Factor Xa	They present anti-inflammatory, antiviral, and anticoagulants effects		Al-Horani (2020)

### Modulating the Expression and Activity of TMPRSS2

Besides SARS-COV, the TMPRSS2 is critical for viral spread of the H3N2 influenza A virus (IAV) and mono-basic H1N1 virus (Hatesuer et al., 2013) and also for the replication and pathogenesis of the H10 subtype of IAV in mice (Lambertz et al., 2019), where knock-out Tmprss2 mice were resistant to the virus. This subtype can also infect humans emphasizing the significance of the TMPRSS2 for drug development against multiple IAV subtypes (Lambertz et al., 2019). The TMPRSS2 can also activate HCV infection at the entry and post-binding stages and involve in the persistence, pathogenesis, and sensitivity of HCV infection (Nickols and Dervan, 2007). Since the TMPRSS2 is involved in other viral infections such as coronavirus (MERS-CoV, SARS-CoV, hCoV-EMC, and HCoV-229E) (Glowacka et al., 2011; Bertram et al., 2013; Gierer et al., 2013; Shirato et al., 2013), hepatitis C virus (Esumi et al., 2015), and influenza A virus (Shen et al., 2020), it would be an attractive alternative against a wide spectrum of respiratory viruses, especially SARS-CoV-2. Taken together, the TMPRSS2 is a target for antiviral therapy.

As we mentioned earlier, the AREs are involved in the TMPRSS2 expression and can be striking drug targets. A polyamide compound can bind to the ARE in the TMPRSS2 promoter and moderately repress its expression (Nickols and Dervan, 2007). Wang et al. (2020) by mining publicly available data on gene expression, identified that the estrogen-related compounds including genistein, androgen receptor antagonist enzalutamide and estradiol can down-regulate the TMPRSS2. These reports suggest that the aforementioned drugs can be promising therapeutic candidates for the treatment of COVID-19. (Wang et al., 2020b).

The promoter of the human *TMPRSS*2 gene has a guanine-rich region forming G-quadruplex secondary structures that can block or reduce the TMPRSS2 transcription in the presence of potassium ions (Shen et al., 2020). Benzoselenoxanthene analogs could significantly down-regulate the TMPRSS2 expression by stabilizing G-quadruplex structure and could prevent the growth and spread of influenza A virus *in vitro* (Shen et al., 2020). Therefore, the down-regulation of the TMPRSS2 mRNA through G-quadruplex structure stabilizers can be a promising strategy in developing novel small molecule drugs against SARS-CoV-2.

Antioxidants serve as regulators of the protease/antiprotease balance that can prevent viral infection. Antioxidants—the so-called free radical scavengers- are natural or man-made substances that can prevent or neutralize free radicals’ damage to cells. It has been elucidated that the master antioxidant transcriptional factor (Nrf2) could down-regulate the expression of the TMPRSS2 in prostate cancer cell lines, thus causing alternations in the protease/antiprotease components balance and subsequently, and result in protection against the respiratory infections (Meyer and Jaspers, 2015). The Nrf2 has a critical role in the reduction of oxidative stress; besides, it exerts beneficial effects in respiratory epithelial responses to respiratory viral infection. Various types of antioxidants are available nowadays. Sulforaphane (SFN) is a potent antioxidant belonging to the class of isothiocyanates, a sulfur-containing organic compound naturally found in cruciferous vegetables (e.g., cauliflower and broccoli). It may decrease oxidative stress and inflammation besides exerting antimicrobial effects. It has been shown that the beneficial effects of the SFN supplementation depend on promoting the Nrf2, cellular antioxidants such as heme oxygenase-1 (HO-1) and NADPH quinone oxidoreductase 1 (NQO1) activities, thus, in turn, prevents the secretion of pro-inflammatory mediators. The SFN down-regulates TMPRSS2 levels and results in the protection against infection. Available reports elucidated that the Nrf2 negatively regulates the TMPRSS2 (Meyer and Jaspers, 2015). Kesic and colleagues demonstrated that the SFN exerts protective effects against respiratory viruses and reduces the IAV entry into respiratory epithelial cells probably as a result of a reduction in TMPRSS2 expression (Kesic et al., 2011).

In addition to the possible androgen receptor-targeted treatments to modify the expression of the TMPRSS2, impairing its protease activity would be an alternative approach. Camostat mesylate, known as FOY 305, is clinically used to treat chronic pancreatitis. It could protect cultured lung epithelia and mice from infection with the H1N1 influenza virus (Bahgat et al., 2011). Shirato et al. report that the treatment with camostat (a single dose) could sufficiently hamper the MERS-CoV entry into Calu-3 cells (a lung-derived cell line) and possibly into the lung (Shirato et al., 2013). Moreover, the viral multistep growth was significantly repressed and virus-induced host cell death was hindered. They concluded that the camostat-inhibited TMPRSS2 or other serine proteases may determine virus pathogenesis and tropism in the lung (Shirato et al., 2013). It is also reported that the SARS-CoV pathogenesis and spread can be efficiently prohibited by camostat (Zhou et al., 2015). This drug can target the TMPRSS2 protease and reduce the replication rate of SARS-CoV-2 (Hoffmann et al., 2020c).

Bromhexine hydrochloride (BRH) is a TMPRSS2 inhibitor that can attenuate metastasis in prostate cancer mice models (Lucas et al., 2014). Being an FDA-approved drug in mucolytic cough suppressants with no significant adverse effects, it can effectively be used against coronavirus infections (Barzegar et al., 2020; Wang et al., 2020c; Fu et al., 2020; Tolouian et al., 2020). In this regard, Li et al. conducted a clinical pilot study on Chinese patients to evaluate the beneficial effects of BRH tablets in moderate COVID-19 treatment. Their findings illustrated that BRH enhanced chest computed tomography (CT), the need for oxygen therapy, and the discharge rate within 20 days (Irham et al., 2020). Nafamostat –an anticoagulant- is another drug that can inhibit the activity of the TMPRSS2, so, it can reduce the viral entrance and block MERS-CoV infection *in vitro* (Yamamoto et al., 2016). Recently, evidence from the latest studies showed the advantages of nafamostat in COVID-19 patients (Asakura and Ogawa, 2020; Hoffmann et al., 2020d). A case study performed on three elderly COVID-19 patients in South Korea demonstrated that nafamostat administration leads to disease prevention through regulating the complement cascade and blocking DIC. Furthermore, it may inhibit virus invasion by impeding virus fusion on the cell membrane (Jang and Rhee, 2020). Altogether, the TMPRSS2 has potential therapeutic benefits against respiratory coronavirus infections. Plasminogen activator inhibitor-1 (PAI-1) is an effective membrane-anchored serine protease inhibitor. It can inhibit TMPRSS2-mediated hemagglutinin cleavage and repress the influenza virus in animals.

Mc Cord et al. (2020) discovered that the PB125 could be used as a therapeutic agent in COVID-19 patients, evidenced by upregulated LIF, suppressed inflammatory responses, inhibited *TMPRSS*2 gene expression directly or by prevention of PAI-1, encoded by the SERPINE1 (Dittmann et al., 2015) and increased Nrf2 activity by HDAC5 downregulation (Hu et al., 2019). In the human airway epithelial cell line, aprotinin and synthetic inhibitors of the TMPRSS2 could inhibit the replication of SARS-CoV-2. Combining several inhibitors of the TMPRSS2 could result in a more effective antiviral activity against the virus than a single serine protease inhibitor. The top 12 natural compounds that significantly can interact with the active sites of the TMPRSS2 are reviewed by Rahman et al. (202).

### Furin Inhibitors

Unlike the TMPRSS2, furin and furin-like enzymes are essential for several pathways and normal development, therefore, its prolonged blockade may lead to some adverse and toxic effects (Hasan et al., 2020). However, a brief furin inhibition may exert a therapeutic benefit and be tolerated (Sarac et al., 2002).

To achieve satisfactory outcomes, a mixture of protease inhibitors would be required. The combination of furin inhibitors that target different proteases of SARS-CoV-2 might be an interesting therapeutic strategy (Wu et al., 2020b). Moreover, a combined administration of furin and the TMPRSS2 inhibitors can be used to target both of these proteases. Evidence shows that the combination of furin inhibitor MI-1851 with several TMPRSS2 inhibitors (MI-1900 and MI-432) could produce more effective antiviral activity against the newly emerged virus than any single serine protease inhibitor (Bestle et al., 2020a).

### Cathepsin Inhibitors

The inhibitors of cathepsin B (CA-074), cathepsin L (SID 26681509), and calpain (E64D) were tested in HEK 293/hACE2 cells. The findings illustrated that 293/hACE2 cells treated with E64D, had a decreased entry of the SARS-CoV2 S pseudovirions (about 92.5%), which in turn highlight the role of at least one of the calpain or cathepsins for SARS-CoV-2 entry. Moreover, treatment with cathepsin L inhibitor by 76% reduced the entry of SARS-CoV-2 S pseudovirions, indicating the probable role of lysosomal cathepsin L in priming SARS-CoV-2 S protein in 293/hACE2 cells. However, cathepsin B inhibitor showed no significant effects on virus entry (Korkmaz et al., 2020; Sahebnasagh et al., 2020). Applying both the E-64D and camostat mesylate results in a complete inhibition of SARS-CoV-2 S protein-driven entry into Vero-TMPRSS2 cells and Caco-2 cells which proposed that both cathepsins B, L and TMPRSS2 is required for priming cells (Hoffmann et al., 2020a). The inhibition of cathepsin L offers two possible steps for the coronavirus infection, blocking virus entry on the host cell surface and viral material release and replication inside the host cell endosomes (Liu et al., 2020).

### Other Protease Inhibitors

Utilizing sivelestat- a neutrophil elastase inhibitor- leads to acute lung injury (ALI) alleviation *via* enhancing alveolar epithelium, vascular endothelium injuries and reducing vascular permeability induced by neutrophils. Though it can be considered as a novel treatment approach in controlling ALI/ARDS or coagulopathy in COVID-19 patients (Sahebnasagh et al., 2020). The suppression of plasmin activity by antiproteases may prevent SARS-CoV-2 entry into respiratory cells and alleviate the clinical outcomes of COVID-19 patients (Ji et al., 2020).

## Clinical Trials

Ansarin et al. for the first time performed an open-label randomized clinical trial, to investigate the efficacy of early administration of bromhexine in patients with COVID-19 pneumonia in Tabriz, Iran. The oral administration of bromhexine results in a significant reduction in ICU admissions, intubation and mortality in the treated group in comparison to the control group. Furthermore, bromhexine treatment exhibited an improvement in C-reactive protein (CRP), lactate dehydrogenase (LDH), and neutrophil/lymphocyte ratio (NLR) levels within two weeks when compared to the control group. However, their results demonstrated that there was no significant difference in the length of hospital stay among treated and control groups (Ansarin et al., 2020).

Li and colleagues conducted an open-label randomized controlled pilot study to examine the efficacy and safety of bromhexine hydrochloride in the treatment of moderate COVID-19. The oral administration of bromhexine hydrochloride (for 14 consecutive days) improved chest computed tomography, a need for oxygen therapy, and discharge rate in 20 days. Nevertheless, their data were not statistically significant (Shang et al., 2020).

Hofmann-Winkler et al. evaluated the efficacy of camostat mesylate in critically ill COVID19 patients with organ failure admitted in ICU of University Hospital Göttingen, Germany. Their findings revealed that the Sepsis-related Organ Failure Assessment score declined in the camostat mesylate-treated group; however, in the hydroxychloroquine group it remained high. Besides, camostat mesylate administration results in a reduction of disease severity, inflammatory markers and amelioration of oxygenation within 8 days in comparison to patients receiving hydroxychloroquine (Hofmann-Winkler et al., 2020). Doi et al. reported the efficacy of nafamostat mesylate in combination with favipiravir in critically ill COVID19 patients admitted to the ICU at The University of Tokyo Hospital. Nafamostat therapy targets the virus entry in host epithelial cells and impedes intravascular coagulopathy (Doi et al., 2020).

## Conclusion

Unique landscapes of SARS-CoV-2 entry are low frequency of RBD standing up that is implicated for immune evasion; moreover, its RBD has a high binding affinity to hACE2 that provides an efficient entry. Finally, the pre-activation of the spike by furin enhances viral entry into some cells. All these features compensate for SARS-CoV-2’ hidden RBD and possibly permit the virus to preserve an effective cell entry yet evading immune surveillance. All these characteristics may be responsible for the viral widespread.

Among different host factors that are involved in the entrance of SARS-CoV-2, transcriptional inhibition of the TMPRSS2 seems to be a hopeful strategy. As with scientific coincidences, our TMPRSS2 insights are obtained from cancer research. TMPRSS2 inhibitor could decrease prostate cancer severity in a mouse model. Additional evidence for effective inhibition of the TMPRSS2 comes from a report indicated that aprotinin could successfully inhibit influenza virus infections (Zhirnov, 1987), although the HA activating protease TMPRSS2 was unknown at that time—thereafter identified by Böttcher et al. (2006). Since the TMPRSS2 is vital for SARS-CoV-2 entry into the host cells, we propose that the same TMPRSS2 inhibitors may decrease or prevent SARS-CoV-2 infection and can be an affordable medicine in blocking the TMPRSS2. The results of our clinical trial supported this idea and indicated that the administration of bromhexine is promising in the early stage of the COVID-19. It should be noted that transcriptional inhibition of the TMPRSS2 may not be destructive since it seems the TMPRSS2 has no important role in any organ and its blockade does not compromise normal development and homeostasis in the host. However, there are conflicting data on the role of TMPRSS2 blocking in the prevention and/or treatment of COVID-19. Well-designed and large-scale clinical trials are required to shed light on this issue in clinical practice.
